# Supplementary material for: Human Umbilical Cord Mesenchymal Stem Cell-Derived Exosomes Rescue Testicular Aging
Source: Biomedicines. 2024 Jan 3;12(1):98. doi: 10.3390/biomedicines12010098 (PMC10813320; doi:10.3390/biomedicines12010098)
Supplement: Supplementary file 1 [file biomedicines-12-00098-s001.zip › Table S1.pdf]

Table S1. Antibodies used for immunofluorescent staining and western blot.

Antibodies		
IgG mouse monoclonal anti-CD9 (WB 1:1000)	Abcam	Cat#ab236630
IgG mouse monoclonal anti-TSG101 (WB 1:1000)	ABclonal	Cat#A2216
IgG mouse monoclonal anti-CALNEXIN (WB 1:1000)	ABclonal	Cat#A4846
IgG rabbit polyclonal anti-LAMINB1 (IF 1:200)	GeneTex	Cat#GTX03695
IgG rabbit polyclonal anti-PCNA (IF 1:200)	GeneTex	Cat#GTX100539
IgG rabbit polyclonal anti-DDX4 (IF 1:400)	GeneTex	Cat#GTX116575
PNA (IF 1:400)	Sigma-Aldrich	Cat#L7381
IgG rabbit polyclonal anti-STRA8 (IF 1:200)	Thermo Fisher	Cat#PA5-115977
IgG mouse polyclonal anti-CYP17A1 (IF 1:200)	GeneTex	Cat#GTX56294
IgG rabbit polyclonal anti-HSD3B1 (IF 1:200)	Thermo Fisher	Cat#PA5-119789
IgG Goat polyclonal Anti-rabbit, Alexa 594 (IF 1:1000)	Thermo Fisher	Cat#A-11001
IgG Goat polyclonal Anti-mouse, Alexa 488 (IF 1:1000)	Thermo Fisher	Cat#A-11029
IgG Goat anti-mouse Cross-Adsorbed Secondary Antibody (WB 1:1000)	Thermo Fisher	Cat#G-21060

Abcam (Boston, MA, USA); ABclonal (Wuhan, China); GeneTex (Alton Pkwy, Irvine, CA, USA); Sigma-Aldrich (St. Louis, MO, USA); Thermo Fisher (Waltham, MA, USA).
