# Supplementary material for: Human Umbilical Cord Mesenchymal Stem Cell-Derived Exosomes Rescue Testicular Aging
Source: Biomedicines. 2024 Jan 3;12(1):98. doi: 10.3390/biomedicines12010098 (PMC10813320; doi:10.3390/biomedicines12010098)
Supplement: Supplementary file 1 [file biomedicines-12-00098-s001.zip › Table S2 .pdf]

Table S2. Primers used for qRT-PCR analysis.

Gene	Forward Primer	Reverse Primer
Dazl	ATGTCTGCCACAACTTCTGAG	CTGATTTCGGTTTCATCCATCCT
Uchl1	GATGCTGAACAAAGTGTTGGC	GGAGTTTCCGATGGTCTGCTT
Utf1	TGTCCCGGTGACTACGTCT	CCCAGAAGTAGCTCCGTCTCT
Sycp3	AGCCAGTAACCAGAAAATTGAGC	CCACTGCTGCAACACATTCATA
Tex101	ATCTTTCTTCTAATCGCCTCACG	GCTCAGCCTTTGAAGTCCAGT
Pttg1	TCTGATCCGCTGTACTCTCCT	AGGCGGCAATTCAACATCCA
Acrv1	TCAGCAACTTTCAAGCGAGTAT	CTCCTGAAGAGTGCTCACCTG
Tssk1	CTCAAGCGACGAGGCTACATC	ACCGCCACGTTGAACTTTAGG
Spaca1	CCCGAGTCCGAAACCACAG	ACAACACACTTGGATTCACCTC
Tnp2	TCACACCAGTAACCAGTGCAA	CCCTGAGCTACGCCTCTTAG
Prm2	ATGGTTCGCTACCGAATGAGG	CTCCGCCTTCTGCATGACC
Best1	ACACAACACATTCTGGGTGC	CGCAAAGTACACACCTCATTCA
Star	ATGTTCCTCGCTACGTTCAAG	CCCAGTGCTCTCCAGTTGAG
Hsd3b1	TGGACAAAGTATTCCGACCAGA	GGCACACTTGCTTGAACACAG
Cyp11a1	AGGTCCTTCAATGAGATCCCTT	TCCCTGTAAATGGGGCCATAC
Cyp17a1	GCCCAAGTCAAAGACACCTAAT	GTACCCAGGCGAAGAGAATAGA
Hsd17b3	AGGTTCTCGCAGCACCTTTTT	CATCGCCTGCTCCGGTAATC
Nr5a1	CCCAAGAGTTAGTGCTCCAGT	CTGGGCGTCCTTTACGAGG
Insl3	TCCTGGCTATGTCATTGC	TGTGGTCCTTGCTTACTG
Il-1b	GAAATGCCACCTTTTGACAGTG	TGGATGCTCTCATCAGGACAG
Il-6	AGTCCGGAGAGGAGACTTCA	ATTTCCACGATTTCCCAGAG
Tnf	CAGGCGGTGCCTATGTCTC	CGATCACCCCGAAGTTCAGTAG
Ccl2	TTAAAAACCTGGATCGGAACCAA	GCATTAGCTTCAGATTTACGGGT
Ccl7	CCACATGCTGCTATGTCAAGA	ACACCGACTACTGGTGATCCT
Nos2	GTTCTCAGCCCAACAATACAAGA	GTGGACGGGTCGATGTCAC
Il-4	GGTCTCAACCCCCAGCTAGT	GCCGATGATCTCTCTCAAGTGAT
Il-10	CTTACTGACTGGCATGAGGATCA	GCAGCTCTAGGAGCATGTGG
Ccl17	TACCATGAGGTCACTTCAGATGC	GCACTCTCGGCCTACATTGG
Gapdh	AGGTCGGTGTGAACGGATTTG	TGTAGACCATGTAGTTGAGGTCA
