# Supplementary figures and images for: Human Umbilical Cord Mesenchymal Stem Cell-Derived Exosomes Rescue Testicular Aging
Source: Biomedicines. 2024 Jan 3;12(1):98. doi: 10.3390/biomedicines12010098 (PMC10813320; doi:10.3390/biomedicines12010098)

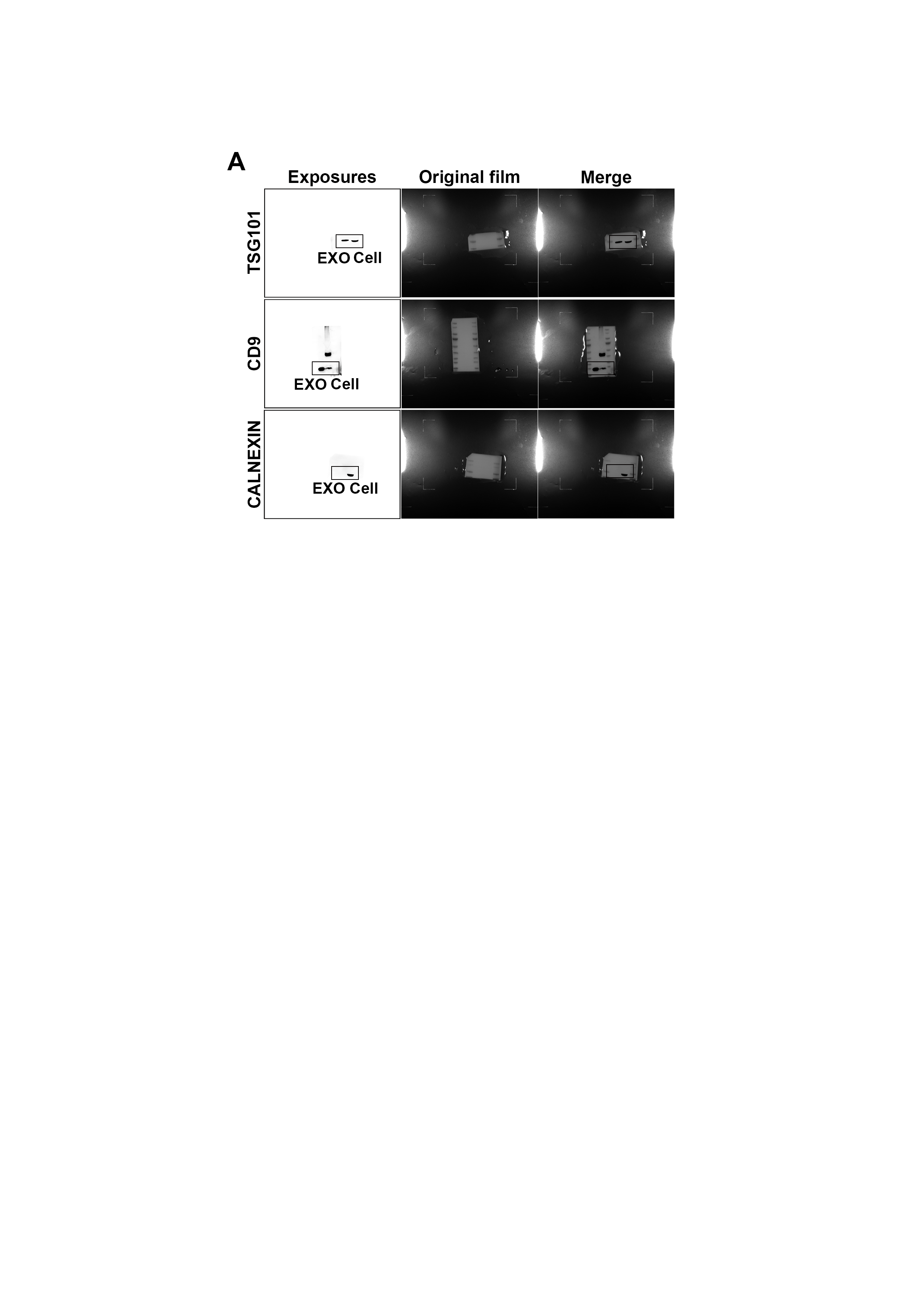

Supplement: Supplementary file 1 [file biomedicines-12-00098-s001.zip › Figure S1.tif]

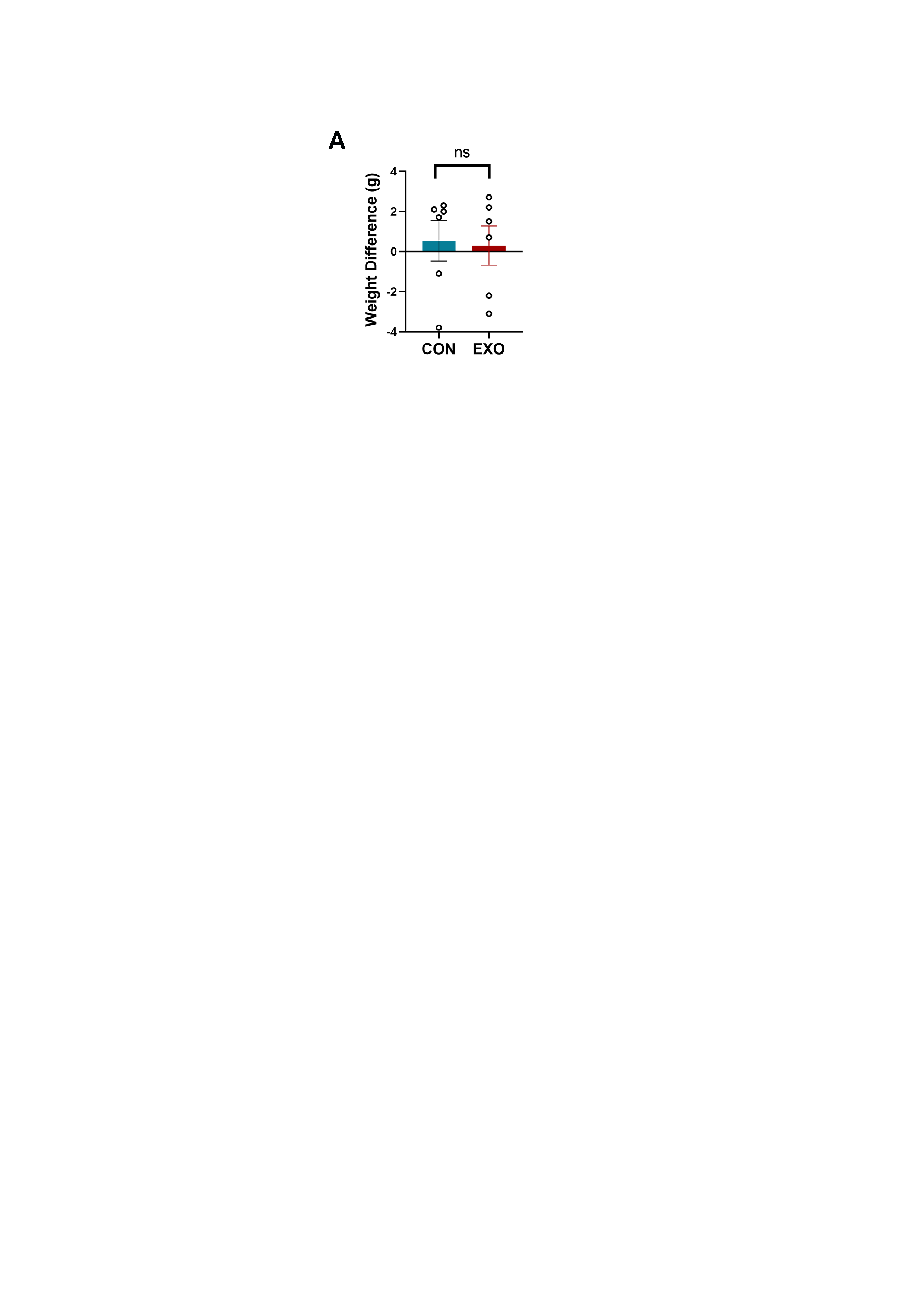

Supplement: Supplementary file 1 [file biomedicines-12-00098-s001.zip › Figure S2.tif]
